# A Manual of Syphilis and the Venereal Diseases

**Published:** 1901-08

**Authors:** 


A Manual of Syphilis and the Venereal Diseases.—By James Nevins Hyde, A. M., M. D., Professor of Skin, Genito-Urinary and Venereal Diseases, Rush Medical College, Chicago, etc.; and Frank Hugh Montgomery, M. D., Associate Professor of Skin, Genito-Urinary and Venereal Diseases, Rush Medical College, Chicago, etc. Second edition; revised and enlarged, with 58 illustrations in the text and 19 full-page lithographic plates; 594 pages. Price, $4.00. Philadelphia: W. B. Saunders & Co. 1900.

The authors intended that this work should be mostly suited to students and the general practitioners. The expert in these lines can afford to discuss the controverted points, hoping in time that crystals will fall which can be utilized in the revision of such works as this one. A long experience in teaching these branches qualifies the authors for getting out an eminently practical book, and from the reviewer's standpoint of view a great success has been attained.

The work has been before the profession long enough to establish it as a standard. It is a text-book in many of the schools, and this department highly commends it. T. J. B.

